# The Story of the Insane from Year to Year

**Published:** 1895-08-24

**Authors:** 


### THE STORY OF THE INSANE FROM YEAR TO YEAR.

EIGHTH SERIES.

### NORWICH BOROUGH ASYLUM.

Here the numbers were almost stationary, being 302 on January 1st, and 304 on December 31st. The admissions were 103, the discharged recovered, 25; relieved, 28; and the deaths were 28. The recoveries work out at 38·2 per cent. on the admissions, and the deaths at 10·7 on the average number resident. Fourteen of the deaths were due to cerebral diseases, 4 to phthisis, and 1 to enteritis. Hereditary influence caused the insanity in 15 of the admissions, intemperance in drink in 9, and mental anxiety in 4. The amount of useless certificating and reporting now common in English asylums is really assuming absurd dimensions. Here, with only 300 patients, Dr. Harris had, during 1894, to make 352 certificates, "not including further reports of other cases to the Commissioners in Lunacy. For 28 patients no less than 140 certificates of death were given by law, in addition to others given to relations or friends." The reader may form some idea of what takes place in those asylums where the patients are numbered by the thousand. The committee "place on record their unqualified confidence in Dr. Harris, and their high appreciation of his work and worth." The weekly cost per head is nearly 9s. 6d.

### DERBY COUNTY ASYLUM AT MICKLEOVER.

On January 1st, 1894, this asylum contained 450 patients, and by the end of the year the number had risen to 464. There were 162 admissions, 66 recoveries, and 45 deaths. The percentage of recoveries on admissions is 42·3, and that of the deaths on the average resident is 9·7. Of the deaths, 23 were from cerebral or spinal diseases, 6 from consumption, and 1 from peritonitis. Hereditary tendency caused the insanity in 41 of the admissions, intemperance in drink in 14, and domestic trouble in 16. Dr. Lindsay strongly advocates the advisableness of building accommodation for private patients, and to show what has been done in this matter he instances the asylums at Dorchester, Cornwall, Cumberland, and the new London Asylum at Claybury. There can be no doubt that such accommodation is needed in almost every county in England, and it will soon be a very pressing question, because the last Lunacy Act does not permit the licensing of any more private asylums, and with the increased population of the country these asylums will be filled to the utmost. The great objection to the erection of blocks for private patients at our county asylums is that the new Lunacy Act and the requirements of the Lunacy Commissioners have so enormously increased the clerical work of medical officers, that if things be not altered soon, there will be great risk of these medical men degenerating into bookkeepers and report-writers. Dr Lindsay would do well to think over this aspect of the question before he builds at Mickleover. We are much pleased to note that one of the night attendants obtained a pension of £37 a year, being twenty-two fortieths of his wages. A pension scheme has at last been adopted by the visitors of this asylum, and it is not surprising that "the adoption of a fixed scale of pension by the committee of visitors has given much satisfaction to the staff, who are grateful to the committee for their sympathetic consideration and attention to this important matter." Important it certainly is—in fact, it is a matter of life or death to many. We earnestly advise every member of the staff to join the National Pension Fund for Nurses, and so increase their provision for old age. The weekly cost of maintenance per head is 9s. 8⅝d.

